# Cardiorespiratory and antinociceptive effects of two different doses of lidocaine administered to horses during a constant intravenous infusion of xylazine and ketamine

**DOI:** 10.1186/1746-6148-9-199

**Published:** 2013-10-09

**Authors:** Pedro I Nóbrega Neto, Stelio PL Luna, Patricia Queiroz-Williams, Khursheed R Mama, Eugene P Steffey, Adriano B Carregaro

**Affiliations:** 1 Academic Unit of Veterinary Medicine, Health and Rural Technology Center, Campina Grande Federal University (HRTC/CGFU), Patos, Paraíba, Brazil; 2 Department of Veterinary Surgery and Anesthesiology, School of Veterinary Medicine and Animal Science, UNESP–Univ Estadual Paulista, Botucatu, Botucatu, São Paulo 18618970, Brazil; 3 Department of Veterinary Clinical Sciences, School of Veterinary Medicine, Louisiana State University, Baton Rouge, LA 70803, USA; 4 Department of Clinical Sciences, College of Veterinary Medicine and Biomedical Sciences, Colorado State University, Fort Collins, CO 80523-1678, USA; 5 Department of Surgical and Radiological Sciences, University of California, Davis, CA 95616, USA; 6 Department of Veterinary Medicine, University of São Paulo, Pirassununga, SP 13635-900, Brazil

**Keywords:** Anesthesia, Intravenous, Bispectral index, Electroencephalography, Antinociception, Analgesia

## Abstract

**Background:**

This study investigated the antinociceptive effects of a constant rate infusion (CRI) of lidocaine during xylazine and ketamine anesthesia in horses and aimed to correlate these effects with cardiorespiratory variables, bispectral index (BIS) and plasma lidocaine concentrations. Six adult crossbred mares weighing 320–400 kg were anesthetized on three different occasions. Sedation was performed with xylazine (0.75 mg/kg IV) and anesthetic induction with guaifenesin (75 mg/kg IV) and ketamine (2 mg/kg IV). Anesthesia was maintained with 37.5 μg/kg/min of xylazine and 87.5 μg/kg/min of ketamine both administered intravenously for 75 min. The three treatments consisted of: lidocaine (loading dose: 5 mg/kg, CRI: 100 μg/kg/min; THL); lidocaine (loading dose: 2.5 mg/kg; CRI: 50 μg/kg/min: TLL); and saline (TS); all given 15 min after induction and maintained for 1 h. Antinociception was measured by response to electrical stimulation and bispectral index (BIS) was recorded during anesthesia. Parametric and non-parametric data were compared using ANOVA followed by Student-Newman-Keuls and Friedman tests, respectively.

**Results:**

Plasma lidocaine concentrations peaked at the end of lidocaine loading dose and was greater in THL (9.61 ± 2.75 μg/mL) vs TLL (4.50 ± 3.34 μg/mL). Electrical noxious stimulation caused purposeful movement in all horses from TS, but no response in THL. The BIS was decreased in THL only and was less when compared to the other treatments throughout anesthesia. Blood pressure, PaO_2_ and PaCO_2_ increased and heart rate (HR), respiratory rate (RR), pH, total plasma protein and temperature decreased during anesthesia in all treatments. PaCO_2_ and HR were greater and RR and pH less in THL compared to TLL and TS at 30 min during anesthesia. All recoveries were considered excellent. Time to standing was longer after THL (60 ± 20 min) than following TLL and TS (32 ± 17 and 30 ± 15 min, respectively).

**Conclusions:**

At the highest dose administered (THL) lidocaine CRI during xylazine/ketamine anesthesia decreased BIS and motor response to noxious stimulation, and prolonged recovery time without significant added cardiorespiratory depression.

## Background

The continuous intravenous (IV) infusion (CRI) of the combination of α_2_-adrenoceptor agonists and ketamine has been extensively studied in horses [1-7]. These combinations may provide less cardiorespiratory depression with no endocrine stress response when compared to inhalation anesthesia [2,4,5,8]. Also, the combination of xylazine and ketamine produces a mild increase in arterial blood pressure and a reduction of the respiratory rate and respiratory acidosis [3,9].

Lidocaine has a number of beneficial effects when administered IV or intramuscularly (IM). It produces systemic analgesia [10-13] and reduces the dose of thiopentone necessary to produce hypnosis in humans [14], dogs [15] and goats [16]. Also, lidocaine constant rate infusion (CRI) reduced the MAC of halothane in both, dogs and horses, from 10% to 45% and 30% to 70%, respectively [10,17]. It reduced MAC of isoflurane by 25% in horses [18], 18.3% in goats [19], and MAC of sevoflurane by 26.7% in horses [20].

The BIS is a number produced by decomposition of the electroencephalogram (EEG) recording and it is quantified by the level of the synchronization signal with the amplitude and frequency (bispectral analysis). Therefore, it is a simplified description of the complex EEG patterns in only one number [21]. In humans a BIS score of 100 means a completely conscious (alert) patient, a score of 70 means a discreet consciousness, and numerical values of 60, 40, and 0 correspond to moderate, deep and very deep hypnosis [21,22]. The BIS is used for measuring the degree of hypnosis during anesthesia [21,23]. Systemic lidocaine levels decrease BIS and are able to suppress it to 0 [24].

Based on the hypothesis that a CRI of lidocaine would potentiate antinociception and decrease BIS without compromising cardiorespiratory variables, the objective of this study was to investigate the anesthetic effects of a CRI of lidocaine during xylazine and ketamine anesthesia in horses and to correlate these effects with cardiorespiratory variables, BIS and plasma lidocaine concentrations.

## Methods

This study was approved by the Institutional Research Animal Care Committee. Six clinically healthy cross-bred Mangalarga Paulista mares, from 5 to 12 (8 ± 3) years of age and weighing between 320 and 400 kg (356.5 ± 31) were used. Animals were provided by the University farm. They were administered gastrointestinal anti-parasitic medication (Eqvalan pasta–Merk, Sharp & Dohme Farm. e Vet. Ltda.) two weeks before the study and housed under field conditions eating *Coast-cross* hay and commercial concentrated feed. Each animal was anesthetized on three different occasions using a randomized crossover design, with a washout period of two weeks between treatments. The investigator responsible for scoring muscle relaxation and recovery was blinded to the treatments.

Xylazine/ketamine infusion doses used in this study came from a pilot study involving ten other horses to establish the minimal infusion rate capable to maintain animals immobilized [25]. Immediately prior to study, horses were housed in stalls and food was withheld for 12 hours prior to anesthesia; water was always available. On the morning of the study, animals were weighed and placed in standing stocks for jugular placement of a 14G catheter (Insyte, Becton Dickinson Ind. Cir. Ltda.) using standard aseptic technique. Animals were sedated with 0.75 mg/kg of xylazine IV (Sedazine 10%, Fort Dodge Saude Animal Ltda.), and 5 minutes later anesthesia was induced with 75 mg/kg of 10% guaifenesin IV (Eter gliceril guaiacol, Henrifarma Prod. Quim. e Farm. Ltda) and 2 mg/kg of ketamine IV (Vetaset 10%, Fort Dodge Saude Animal Ltda). Immediately after anesthetic induction, the animals received a constant infusion of 37.5 μg/kg/min of xylazine and 87.5 μg/kg/min of ketamine. Animals were orotracheally intubated, placed in left lateral recumbency, and connected to a large animal circle anesthetic machine (VML Anesthesia Machine, Matrix Medical Inc.) without a vaporizer in the circuit. The circle system was previously washed out with a flow of 5 L/min of O_2_ over five minutes to ensure an inspired O_2_ concentration (Monitor Cardiocap 5, Datex Ohmeda) close to 100%. After the horse’s endotracheal tube was connected to the anesthetic circuit, the O_2_ flow was adjusted to 10 mL/kg/minute and a gas analyser (Cardiocap 5, Datex Ohmeda) was used to confirm the absence of any volatile anesthetic in the circuit. A 20G catheter was placed in the transverse facial artery for arterial blood pressure measurement and arterial blood sampling.

Fifteen minutes after induction one of three treatments was randomly administered as follows:

Treatment 1 (THL): 5 mg/kg bolus of lidocaine (Xylocaina 2%, Astra Química e Farmacêutica Ltda.) was administered IV over 5 minutes, followed by a CRI of 100 μg/kg/min.

Treatment 2 (TLL): 2.5 mg/kg bolus of lidocaine (Xylocaina 2%, Astra Química e Farmacêutica Ltda.) was administered IV over 5 minutes, followed by a CRI of 50 μg/kg/min. Lidocaine was diluted to 1% with normal saline (0.9% NaCl) to ensure the same volume of injection as THL.

Treatment 3 (TS): Normal saline (0.9% NaCl) was administered at the same volume as the other two treatments following the same administration procedure. Over the course of study each horse received all three treatments.

The bolus and CRI were both administered via peristaltic pump (LF 2001, Lifemed Pesquisas Médicas Ind. Com. Ltda.). All infusions were stopped 75 minutes after induction and the animals were positioned on the recovery room floor, in the same lateral recumbency as during anesthetic period. They were then extubated and continuously observed until they stood without assistance.

### Measurements

Heart rate (HR) was monitored via continuous ECG using a base-apex lead placement configuration. Arterial blood pressure (ABP) was measured by a transducer calibrated against a mercury column before the beginning of each experiment connected to the transverse facial artery catheter (Cardiocap 5, Datex Ohmeda). Respiratory rate was monitored by a tubing adapted to the “Y” of the circle circuit during anesthesia (Cardiocap 5, Datex Ohmeda). The analyzer was calibrated before each experiment with a standardized calibration gas mixture supplied by the manufacturer (Quick Cal Calibration Gas; Datex-Ëngstrom, Finland). The altitude of the location of the study was 870 m.

Arterial blood gases, plasma glucose, and plasma lactate were measured from heparinized blood samples collected from the transverse facial artery (RapidLAB 865, Chiron Diagnostics). All samples were placed on ice and measured within two hours after collection. Total plasma protein (TPP) was measured using refractometry (Refractometer ATAGO–SPR T2). Rectal temperature (T°C) was monitored by using a digital clinical thermometer (Becton Dickinson Ind. Cir. Ltda.).

The BIS values were measured using cutaneous electrodes placed on the frontal and temporal cephalic region and connected to a BIS monitor (A-2000 Bispectral Index Monitor, Aspect Medical Systems, Inc.).

Muscle relaxation was evaluated using a score from 0 to 3 according to the following scale: 0 = muscle relaxation of trunk and limbs; 1 = muscle tremors in some regions of the trunk and limbs; 2 = muscle tremors in most parts of the trunk and limbs; 3 = rigidity of most parts of the trunk and limbs.

All measurements were performed immediately before xylazine administration (T0), and at 5 (T5), 15 (T15), 20 (T20), 30 (T30), 45 (T45), 60 (T60), and 75 (T75) minutes after induction of anesthesia, except for ABP and muscle relaxation which were not measured at T0. Blood gases, TPP, rectal temperature, and biochemical parameters were not measured at T20. BIS was not measured at T0 and was further evaluated five minutes after the end of anesthesia (T80).

To evaluate antinociception, a standardized electrical stimulus (GRASS S-48 nerve stimulator) of 50 V, 5 Hz, 10 ms was applied via electrodes connected to 0.80 × 30 (21G × 1 ¼) metallic needles introduced in the subgengival area two cm parallel to the molars. The stimulus was applied until there was associated purposeful movement of head, neck, or limbs or for a full 60 seconds if no purposeful movement occurred. The stimulus was applied at T15, T30, T60, and T75 after all measurements and blood samples had been collected for that time point.

Lidocaine plasma concentration was measured by high performance liquid chromatography (HPLC Sistem LC-10VP, Suimadzu Corporation) according to the method described by Doherty & Frazier (1998). Blood samples for lidocaine analysis were collected from the transverse facial artery at T0, T20, T30, T45, T60, T75 and T80 and placed in heparinised tubes. The plasma was harvested and stored at −80°C until analysis. At analysis time plasma samples were defrosted and homogenized. A plasma sample of 1 mL was mixed to 50 μl of trimetoprim (internal standard, 25 μg/mL). Sodium hydroxide (200 μl, 1 M) and methylene chloride (4 mL) were added to each sample, vortexed for 15 minutes, and centrifuged for 2000 rpm for 15 minutes. The sediment was reconstituted with 1 mL of mobile phase (isocratic mixture of 0.03 mol/L potassium dihydrogen phosphate:acetonitrile - 87:13 v/v) and aliquots of 180 μl were injected in the chromatograph (LC-10VP, Suimadzu Corporation), using a 2 mL/min flow and ultraviolet absorbance at 205 nm. Recovery ranged from 82% to 100%. Intra- and inter-assay variability was 2.7% and 4.5% respectively. The limit of detection was 0.031 μg/mL.

Recovery from anesthesia was observed and classified according a previously cited [8] simple descriptive scale where 5 = excellent–no struggling, standing at the first attempt, 4 = good–slight ataxia, but good stability, 3 = tolerable–some ataxia when standing, two or three unsuccessful attempts to stand, 2 = bad–excitement, paddling when in recumbency, marked ataxia with possible fall, and 1 = very bad–excitement during recumbency, many unsuccessful attempts to stand, ataxia and falls after standing, risk of trauma. Time from the end of anesthesia to standing was recorded.

### Statistical method

Statistical analysis was performed with a commercial software program (GraphPad InStat, GraphPad Software Inc.). The parametric data were compared using analysis of variance for repeated measures followed by Student-Newman-Keuls Test. The non-parametric data were compared using analysis of variance followed by Friedman test. The level of significance was set at 0.05. Continuous data are presented as mean ± standard deviation (SD).

## Results

**Figure 1:**
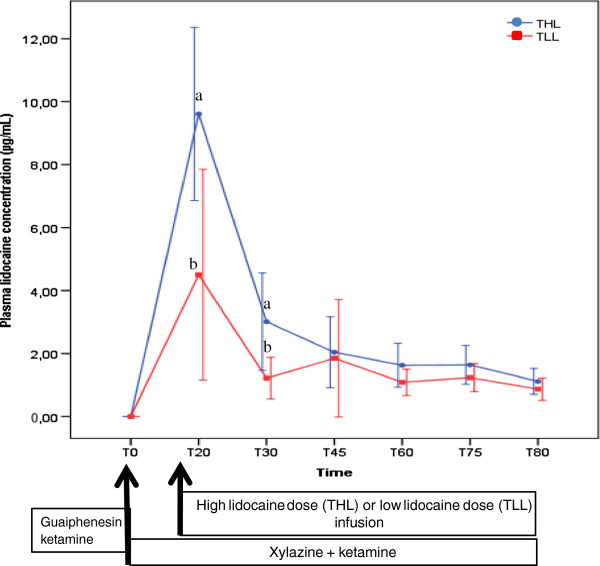
**Mean (SD) plasma lidocaine concentration in mares undergoing intravenous anesthesia with xylazine, guaifenesin and ketamine combined with a high (THL) or low lidocaine (TLL) dose infusion.** a, b - statistical difference between treatments at a given time point.

Maximum plasma lidocaine concentrations were achieved at T20 in THL and TLL and were 9.61 ± 2.75 and 4.50 ± 3.34 μg/mL respectively (Figure 1). Plasma lidocaine concentration was significantly greater in THL versus TLL at 20 and 30 minutes of constant intravenous infusion.

The number of moves versus no moves in response to noxious stimulation for the three groups of horses is given in Table 1. In the absence of lidocaine, 20 out of 24 noxious stimulations caused purposeful movement (TS). Conversely, there was no response to stimulation during the administration of high dose lidocaine (THL). There was no difference in muscle relaxation over time or among treatments. The average scores ranged between 0 and 1.2 ± 0.8. Spontaneous muscular movement (sometimes seizure-like) was observed in the limbs, head, and neck of all THL and in two of TLL horses. This was observed at 3.0 ± 1.1 minutes after the lidocaine loading bolus and starting the lidocaine infusion, and continued for 3.2 ± 1.3 minutes thereafter.

**Table 1:** Response to noxious stimulation (move/no move) and mean (±SD) bispectral index (BIS) values in mares undergoing intravenous anesthesia with xylazine, guaifenesin and ketamine combined with a high lidocaine dose (THL), low lidocaine dose (TLL) or saline infusion (TS) (n = 6)

**Parameter**	**Treat**^**1**^	**Time (minutes)**							
		**T5**	**T15**	**T20**	**T30**	**T45**	**T60**	**T75**	**T80**
Response to noxious stimulation	THL		6/0		0/6*		0/6*^a^	0/6*^a^	
	TLL		4/2		2/4		2/4^ab^	2/4^ab^	
	TS		4/2		4/2		6/0^b^	6/0^b^	
BIS	THL	97 ± 1	96 ± 4	63 ± 12^*a^	43 ± 11^*a^	45 ± 12^*a^	56 ± 19^*a^	63 ± 16^*a^	87 ± 15
	TLL	97 ± 1	96 ± 2	79 ± 26^ab^	75 ± 23^b^	84 ± 21^b^	87 ± 15^b^	91 ± 12^b^	97 ± 2
	TS	96 ± 3	92 ± 9	94 ± 4^b^	95 ± 3^b^	98 ± 0^b^	97 ± 1^b^	98 ± 1^b^	98 ± 1

1- Treat = Treatment; * - statistical difference from the baseline value (row) (P < 0.05); different superscript letters indicate difference between treatments at each time point (column) (P < 0.05). For all means n = 6; T5 and T15–5 and 15 minutes after induction of anesthesia with xylazine, guaifenesin and ketamine respectively (and maintenance with xylazine and ketamine); T20, T30, T60, T75 and T80–5, 15, 45, 60 and 65 minutes after high lidocaine dose (THL), low lidocaine dose (TLL) or saline infusion (TS) respectively.

There was a significant reduction in the BIS from T20 to T75 in THL horses (Table 1). There were no significant changes in BIS in TLL and TS horses during anesthesia. Bispectral index values were significantly lower in THL horses compared to the other treatments at all time points from T30 to and including T75. Within 5 minutes of the cessation of lidocaine delivery, the BIS value for THL horses was no longer significantly different from pre-lidocaine values for this treatment, or for TLL and TS horses.

A decrease in heart rate followed anesthetic premedication and induction with all three treatments, and heart rate remained less than pre-drug values for TLL and TS during the 75 minutes of anesthetic maintenance (Table 2). On the other hand, heart rate was not significantly different from pre-drug values in THL horses within 5 minutes of the start of lidocaine administration (T20). There were no cardiac dysrhythmias noted in any treatment. Arterial blood pressure was first measured shortly following anesthetic induction and constant xylazine and ketamine rate infusion. The first systolic, diastolic and mean arterial blood pressure values were similar for each of the three treatments, and they were the lowest of all subsequently determined values within each treatment (Table 2).

**Table 2:** **Mean (±SD) heart rate (HR), systolic, medium and diastolic arterial pressures (SAP, MAP and DAP), PaO**_**2**_**, PaCO**_**2**_**, respiratory rate (RR), pH and base excess (BE) in mares undergoing intravenous anesthesia with xylazine, guaifenesin and ketamine, combined with a high dose of lidocaine (THL), low dose of lidocaine (TLL) or saline infusion (TS) (n = 6)**

**Parameter**	**Treat**^**1**^	**Time (minutes)**							
		**T0**	**T5**	**T15**	**T20**	**T30**	**T45**	**T60**	**T75**
HR (bpm)	THL	41 ± 5	35 ± 3^*^	33 ± 3^*^	39 ± 8	37 ± 4^a^	36 ± 5	36 ± 5	37 ± 5
	TLL	39 ± 5	35 ± 3^*^	34 ± 3^*^	34 ± 2^*^	34 ± 3^*b^	34 ± 3^*^	35 ± 3^*^	35 ± 3^*^
	TS	42 ± 4	35 ± 4^*^	32 ± 2^*^	33 ± 2^*^	32 ± 3^*b^	34 ± 4^*^	34 ± 4^*^	33 ± 4^*^
SAP (mmHg)	THL	-	138 ± 23	154 ± 23^*^	155 ± 17^*^	158 ± 18^*^	176 ± 21^*^	179 ± 20^*^	175 ± 11^*a^
	TLL	-	141 ± 26	160 ± 28^*^	163 ± 29^*^	168 ± 28^*^	177 ± 30^*^	173 ± 22^*^	175 ± 19^*a^
	TS	-	142 ± 24	168 ± 40^*^	181 ± 42^*^	191 ± 43^*^	190 ± 34^*^	191 ± 26^*^	196 ± 24^*b^
MAP (mmHg)	THL	-	109 ± 17	120 ± 22^*^	123 ± 15^*^	125 ± 14^*^	139 ± 12^*^	146 ± 13^*^	146 ± 13^*^
	TLL	-	113 ± 19	126 ± 22^*^	129 ± 24^*^	132 ± 22^*^	139 ± 21^*^	140 ± 17^*^	144 ± 16^*^
	TS	-	109 ± 19	132 ± 32^*^	142 ± 32^*^	147 ± 32^*^	143 ± 21^*^	146 ± 16^*^	147 ± 14^*^
DAP (mmHg)	THL	-	92 ± 15	101 ± 21	105 ± 14^*^	108 ± 11^*^	118 ± 10^*^	124 ± 12^*^	128 ± 17^*^
	TLL	-	96 ± 14	106 ± 18^*^	110 ± 20^*^	113 ± 19^*^	117 ± 17^*^	120 ± 16^*^	124 ± 19^*^
	TS	-	92 ± 17	113 ± 27^*^	120 ± 27^*^	123 ± 27^*^	119 ± 17^*^	121 ± 14^*^	121 ± 12^*^
PaO_2_ (mmHg)	THL	99 ± 8	266 ± 69^*^	294 ± 74^*^	-	262 ± 81^*^	262 ± 95^*^	261 ± 115^*^	251 ± 102^*^
	TLL	92 ± 3	315 ± 58^*^	319 ± 71^*^	-	305 ± 63^*^	305 ± 67^*^	291 ± 74^*^	290 ± 74^*^
	TS	93 ± 4	256 ± 67^*^	287 ± 62^*^	-	269 ± 61^*^	277 ± 70^*^	284 ± 70^*^	283 ± 75^*^
PaCO_2_ (mmHg)	THL	41 ± 5	55 ± 6^*^	51 ± 5^*^	-	59 ± 5^*a^	55 ± 5^*^	57. ± 5^*a^	56 ± 5^*^
	TLL	44 ± 3	52 ± 3^*^	53 ± 5^*^	-	53 ± 4^*b^	52 ± 4^*^	53 ± 3^*b^	54 ± 3^*^
	TS	43 ± 3	52 ± 5^*^	53 ± 5^*^	-	53 ± 6^*b^	52 ± 6^*^	53 ± 5^*b^	53 ± 6^*^
RR (mpm)	THL	20 ± 7	8 ± 3^*^	10 ± 3^*^	11 ± 5^*^	8 ± 2^*a^	9 ± 2^*^	10 ± 3^*^	10 ± 3^*a^
	TLL	21 ± 4	8 ± 2^*^	10 ± 3^*^	9 ± 1^*^	10 ± 3^*b^	11 ± 2^*^	11 ± 2^*^	13 ± 4^*ab^
	TS	24 ± 7	9 ± 2^*^	8 ± 2^*^	10 ± 2^*^	11 ± 3^*b^	14 ± 9^*^	17 ± 9^*^	16 ± 6^*b^
pH	THL	7.40 ± 0.03	7.34 ± 0.03^*^	7.36 ± 0.02^*^	-	7.32 ± 0.02^*a^	7.35 ± 0.02^*^	7.36 ± 0.02^*^	7.36 ± 0.03^*^
	TLL	7.42 ± 0.01	7.36 ± 0.01^*^	7.36 ± 0.03^*^	-	7.35 ± 0.03^*b^	7.37 ± 0.03^*^	7.38 ± 0.02^*^	7.37 ± 0.02^*^
	TS	7.42 ± 0.03	7.35 ± 0.03^*^	7.36 ± 0.03^*^	-	7.37 ± 0.04^*b^	7.38 ± 0.05^*^	7.38 ± 0.04^*^	7.39 ± 0.04^*^
BE (mmol/L)	THL	1.5 ± 1.7	2.3 ± 1.8	2.3 ± 2.3	-	2.3 ± 2.4	3.6 ± 3.1	4.9 ± 2.1^*^	4.6 ± 1.9^*^
	TLL	3.0 ± 1.5	2.0 ± 1.0	2.9 ± 0.9	-	2.4 ± 1.3	3.0 ± 0.9	4.4 ± 0.4^*^	4.8 ± 1.1^*^
	TS	2.7 ± 1.8	2.0 ± 1.4	3.2 ± 1.0	-	3.8 ± 1.1^*^	4.0 ± 1.5^*^	4.7 ± 1.2^*^	5.2 ± 0.7^*^

1- Treat = Treatment; * - statistical difference from the time 0 or baseline value (row) (P < 0.05); different superscript letters indicate difference between treatments at each time point (column) (P < 0.05). For all means n = 6; T5 and T15–5 and 15 minutes after induction of anesthesia with xylazine, guaifenesin and ketamine respectively (and maintenance with xylazine and ketamine); T20, T30, T60, T75 and T80–5, 15, 45 and 60 minutes after high lidocaine dose (THL), low lidocaine dose (TLL) or saline infusion (TS) respectively.

Arterial blood gas and acid base values are given in Table 2. An increase in PaO_2_ was associated with breathing an oxygen enriched gas mixture during general anesthesia. The PaCO_2_ also increased following induction of anesthesia and remained elevated over the course of anesthesia for all treatments. The values of PaCO_2_ and pH for TLL and TS horses were similar and consistent over the course of anesthesia, while these variables during anesthesia for THL horses were slightly, but generally not statistically significantly, greater (PaCO_2_) and smaller (pH) than that of TLL and TS horses. Respiratory rate was significantly reduced from awake values following anesthetic induction (Table 2).

Average plasma glucose concentration of horses in all three study treatments increased from baseline over the course of anesthesia and reached their highest measured levels of the study at the last sampling point, i.e., T75 (Table 3). Anesthetic induction and maintenance was also associated with an increase in plasma lactate, and small decreases in TPP concentration and temperature (Table 3). Magnitude of initial change and time-related values for each of these four variables was similar for the three study treatments.

**Table 3:** Mean (±SD) plasma glucose, lactate, total plasma protein concentration (TPP) and temperature (T°C) in mares undergoing intravenous anesthesia with xylazine, guaifenesin and ketamine, combined with a high dose of lidocaine (THL), low dose of lidocaine (TLL) or saline infusion (TS)

**Parameter**	**Treat**^**1**^	**Time (minutes)**						
		**T0**	**T5**	**T15**	**T30**	**T45**	**T60**	**T75**
Glucose (mg/dL)	Mean of treatments	103 ± 10	139 ± 22^*^	178 ± 39^*^	213 ± 42^*^	240 ± 44^*^	255 ± 50^*^	266 ± 55^*^
Lactate (mmol/L)	Mean of treatments	0.80 ± 0.25	1.37 ± 0.39^*^	1.51 ± 0.39^*^	1.62 ± 0.40^*^	1.70 ± 0.41^*^	1.73 ± 0.45^*^	1.76 ± 0.50^*^
TPP (g/dL)	Mean of treatments	7.2 ± 0.6	6.9 ± 0.5^*^	6.8 ± 0.5^*^	6.7 ± 0.6^*^	6.7 ± 0.5^*^	6.7 ± 0.5^*^	6.8 ± 0.6^*^
T°C (°C)	Mean of treatments	37.9 ± 0.4	37.9 ± 0.4	37.9 ± 0.4	37.5 ± 0.4	37.3 ± 0.4^*^	37.2 ± 0.5^*^	37.0 ± 0.5^*^

1- Treat = Treatment; * - statistical difference from the time 0 or baseline value (row) (P < 0.05). For all means n = 18; T5 and T15–5 and 15 minutes after induction of anaesthesia with xylazine, guaifenesin and ketamine respectively (and maintenance with xylazine and ketamine); T30, T60, T75–15, 45 and 60 minutes after lidocaine or saline infusion (data from the three groups were grouped).

All recoveries were rated excellent. Time to standing was significantly different between THL (60 ± 20 minutes) horses and those of both TLL and TS (32 ± 17 and 30 ± 15 minutes, respectively). All horses urinated various unmeasured amounts during the anesthetic recovery period both while laterally recumbent, and again within a short time after standing.

## Discussion

The proposed hypothesis that lidocaine infusion would increase antinociception and depth of xylazine/ketamine anesthesia, without affecting cardiorespiratory variables, was confirmed, and justifies the prolongation of anesthetic recovery following the higher bolus dose and infusion rate of lidocaine.

Results of the presently reported study confirm those of previous reports of the IV anesthetic protocol used [9,26], as well as the effects of a 50 μg/kg/min IV infusion of lidocaine during general anesthesia in healthy horses studied both under laboratory [27,28] and clinical [18] conditions. In addition, presently reported results extend current knowledge by offering insight to the use of lidocaine during anesthesia maintained by injectable drugs, as opposed to previous reports of inhalation anesthesia [18,20,28,29], and highlights responses to two different IV doses of lidocaine.

Presently reported circulatory and respiratory system observations recorded over the course of anesthesia time in the absence of lidocaine (i.e., TS horses) are quantitatively similar to those similarly gathered and reported by Mama et al. [9,26] (Table 2). Mama et al. [9] postulated that anesthetic maintenance with the similarly used IV drug regime was at least slightly below the ED_50_ for move/no move responses to a noxious electrical stimulus. The noxious stimulus referred to was the same as that used in the presently reported study, and the one commonly used in determining the minimal alveolar concentration (MAC) for inhalation anesthetics in horses [30]. The 20 moves versus only 4 no move responses in response to the 24 stimulations in the present study (Table 1) are similar (actually a bit less than the earlier study) and these presently reported data are in strong support of the hypothesis voiced earlier by Mama and colleagues.

Bispectral electroencephalographic signal processing has been investigated with human patients in an effort to develop the EEG as a measure of anesthetic depth. In humans, BIS is generally considered to be a good indication of CNS depression. The accuracy of the BIS monitor in quantitating anesthetic depth specifically in the horse has not been rigorously evaluated and reported studies with this focus are very limited [31,32], therefore the results should be considered with caution due to the lack of validity of this technique in these species. However, because of its considerable use with human patients, its empirical trial with species of clinical importance to veterinary medicine, like the horse is logical. The presently reported study provided opportunity to compliment previous reports by gathering additional empirical data on the utility of BIS recordings under controlled conditions in anesthetized horses. Values of BIS in our horses anesthetized without lidocaine infusion were only minimally decreased (Table 1) even immediately following anesthetic induction and were close to the human awake, alert level of 100 (most recordings were 95 or greater). These data support our earlier comments regarding our horses being at a light level of anesthetic recumbency.

Adding lidocaine to the anesthetic protocol (TLL and THL), decreased in dose-related fashion both the BIS and the number of moves (versus no move) in response to noxious stimulation (Table 1) indicating an anesthetic modifying effect of lidocaine (i.e., increased anesthetic depth). BIS values were most reduced with the 100 μg/kg/min rate of lidocaine infusion but there was large variation in BIS values between individual horses. While providing some qualitative measure of support of BIS application to anesthetic management of an individual horse, especially the large between-horse variability raises doubt about its quantitative accuracy in a population of horses. These data thus support previous investigators who questioned the precision of BIS as an indicator of drug-induced CNS depression and therefore minimized the clinical value, of at least present development and understanding, of this technology for anesthetic management of the horse [31,32]. Much more evaluative information (pro or con) is necessary to guide our understanding and conclusions of BIS technology for equine applications.

Using a similar dose in sevoflurane anesthetized horses, like TLL in this study, Rezende and co-workers [20] reported a MAC reduction of 26.7 ± 12%, a value similar to previous reports of lidocaine reduction of halothane [17] and isoflurane [18] anesthetic requirement in ponies and horses respectively. In Rezende et al. study mean plasma lidocaine concentration ranged from a high of 2.6 ng/mL at the end of lidocaine bolus to 2.2 ng/mL at the end of infusion. Except for the initial measurement, these values are similar to those measured in the present study at the 50 μg/kg/min rate (Table 1). The higher initial plasma lidocaine values of the current study likely relate to the higher loading bolus lidocaine injection and the more rapid speed of injection used compared to the sevoflurane MAC study (i.e., 2.5 versus 1.3 mg/kg, respectively, and the 1.3 mg/kg dose administered over 15 minutes). Mean plasma lidocaine concentrations from T30 to the end of infusion were similar to those reported by Rezende and coworkers using a similar IV lidocaine infusion protocol during sevoflurane anesthesia of horses [20].

It is well established that lidocaine may produce convulsions at high intravenous dosage in many species. The lidocaine doses used in this study were below the convulsant dose reported for cats [33], dogs [34,35], rats [36], and monkeys [34]. However, doses were similar to those that caused signs of toxicity in rabbits [33] and humans [34]. The muscle tremors and sometimes forceful limb movements observed in our THL and some TLL horses at the beginning of anesthesia were associated with high plasma lidocaine concentrations likely due to the bolus administration over the short 5 minute time period [33,34,37]. Meyer and colleagues [38] reported skeletal muscle tremors in horses at plasma lidocaine concentrations above 3.24 ± 0.74 μg/mL and, in the present study, the plasma lidocaine concentration obtained at the end of the loading dose (T20) was significantly greater than that. The pro-convulsive nature of ketamine and the hypercapnea present at the time may have further facilitated lidocaine-induced muscle tremors and other limb movements we observed [1,33,34,39]. Doherty & Frazier [17] reported similar plasma lidocaine concentrations in horses anesthetized with halothane. However, muscle tremors were not reported associated with their study. The apparent discrepancy between results of their study and our own may be explained on the basis of a deeper level of anesthesia in the halothane anesthetized horses and associated masking or prevention of the muscle activities. An alternative or additional explanation may be the IV lidocaine loading dose was administered over a longer period of time in the study of Doherty & Frazier [17] (i.e., 15 minutes versus 5 minutes in the presently reported study) and resulted in a lower peak lidocaine plasma concentration.

In agreement with previously reported studies [18,28,29], the addition of lidocaine had little consistent effect on hemodynamic parameters (Table 2). Heart rate decreased from baseline (pre-anesthesia) for all treatment and is presumed reflective of the direct and indirect effects of the xylazine. Other than a tendency of systolic, and early in the course of anesthesia, diastolic and mean arterial blood pressures to be less during lidocaine infusion, there were no dramatic differences between circumstances associated with the presence or absence of lidocaine administration.

Time-matched arterial blood gas and acid base values were similar regardless whether anesthesia was maintained with or without lidocaine (Table 2). PaO_2_ and PaCO_2_ predictably increased in magnitude to acceptable ranges following anesthetic induction and supplemental inspired O_2_. Not surprisingly lidocaine infusion had little effect on PaO_2_ whereas, a modestly increased PaCO_2_ was associated with lidocaine at the high infusion rate (THL), compared to TLL and TS respectively.

Lactate increased over the course of anesthetic time after all treatments similarly to previous studies [8,40-42], implying that lidocaine administration had no important influence on these temporal changes. Anesthetic-induced increase in lactate concentration is commonly considered an indication of anaerobic metabolism or depressed lactate metabolism, as for example, accompanying general anesthesia.

Perhaps our most dramatic finding of this study was the continued temporal increase in blood glucose following preanesthetic medication with xylazine and anesthetic induction, regardless whether or not lidocaine was administered (Table 3). Lidocaine appeared to have no further effect on the magnitude of hyperglycemia. Peak average, maximal values of glucose concentration increased from baseline of 254 and 267%, in TLL and THL respectively at the end of anesthesia. Presumably the increase in plasma glucose concentration relates specifically to the use of the alpha-2 adrenergic agonist drug, xylazine. Alpha-2 agonists diminish insulin release from the pancreas and thus promote hyperglycemia, an action of routine prominence in the horse [43,44]. While the initial rise in glucose was expected, we were somewhat initially surprised by the continued increase in glucose concentration over the course of anesthetic time and drug cocktail administration. To the best of our knowledge the present report is the first such report of temporal increase in glucose concentration accompanying constant infusion of xylazine (in this case 75 minutes). General anesthesia per se is likely not responsible for the progressive plasma glucose increase especially considering the magnitude of rise [45,46]. We presume the progressive rise in glucose is related to the continuous delivery of, in this case, xylazine over the more than 75 minute course of anesthesia. A similar temporal increase in glucose was previously noted during continuous infusion of detomidine-ketamine-guaifenesin to acepromazine premedicated ponies [4].

The biological significance of the prolonged state of hyperglycemia following prolonged alpha-2 agonist infusion in horses remains unknown. Therefore we submit that further investigation of prolonged alpha-2 agonist administration and associated hyperglycemia, especially to identify (or not) any medically adverse effects, is warranted.

The low dose of lidocaine had no effect on post anesthetic recover time compared to anesthesia in the absence of lidocaine infusion, while the high dose increased recovery time. Such behavior is compatible with our earlier described increased anesthetic action noted in THL horses, both presumably related to central sedative effects of lidocaine [17,18,20]. In this study, peri-anesthetic use of lidocaine seemingly contributed neither positively or negatively to the quality of recovery from general anesthesia and the quality of anesthetic recovery in TS horses was similar to that reported by Mama et al. [26].

## Conclusions

Lidocaine infusion during xylazine/ketamine anesthesia produced minor cardiorespiratory depression, and a dose-dependent reduction in BIS and increase in antinociception. Recovery was prolonged only with the higher bolus dose and infusion rate of lidocaine.

## Competing interests

None of the authors has any financial or personal relationships that could inappropriately influence or bias the content of the paper.

The authors declare that they have no competing interests.

## Authors’ contributions

PINN was the main contributor, participated in the design of the study, performed all anesthetic procedures and data gathering, performed the statistical analysis and prepared the final draft of the manuscript. SPLL participated in the design of the study, supervised the animal experiment, revised and submitted the final manuscript. PQW and ABC participated in the study since early phase up to the end of data gathering. They assisted in draft and final manuscript preparation. KRM and EPS participated in study design, and early phase of data gathering. They were involved in interpretation of resultant data and assisted in draft and final manuscript preparation. All authors read and approved the final manuscript.

## Authors’ information

PINN (DVM, PhD); SPLL (DVM, PhD, Diplomate ECVAA); PQW (DVM, MS, PhD); KRM (DVM, PhD, Diplomate ACVA), EPS (DVM, PhD, Charter Diplomate ACVA, Founding Diplomate ECVA), ABC (DVM, PhD).
